# Effect of the Elemental Content of Shells of the Bivalve Mollusks (*Mytilus galloprovincialis*) from Saldanha Bay (South Africa) on Their Crystallographic Texture

**DOI:** 10.3390/biology10111093

**Published:** 2021-10-25

**Authors:** Pavel Nekhoroshkov, Inga Zinicovscaia, Dmitry Nikolayev, Tatiana Lychagina, Alexey Pakhnevich, Nikita Yushin, Jacques Bezuidenhout

**Affiliations:** 1 Frank Laboratory of Neutron Physics, Joint Institute for Nuclear Research, 141980 Dubna, Russia; zinikovskaia@mail.ru (I.Z.); dmitry@nf.jinr.ru (D.N.); lychagina@jinr.ru (T.L.); alvpb@mail.ru (A.P.); ynik_62@mail.ru (N.Y.); 2 Horia Hulubei National Institute for R&D in Physics and Nuclear Engineering, 077125 Bucharest, Romania; 3 Borissiak Paleontological Institute, Russian Academy of Sciences, 117997 Moscow, Russia; 4 Faculty of Military Science, Stellenbosch University, Victoria Street, Stellenbosch 7602, South Africa; Jab@ma2.sun.ac.za

**Keywords:** neutron diffraction, crystallographic texture, pole figures, neutron activation analysis, trace elements, bivalve mollusk shells

## Abstract

**Simple Summary:**

The aim was to study the question of whether the texture of mollusk shells changes with alteration in their elemental composition. Even though significant differences between concentrations of elements among the stations were found, the crystallographic textures of mussel shells from the studied bays showed insignificant dissimilarities. The observed differences in the maximum values in the pole figures fell within the range of variability identified for the genus *Mytilus*. Nevertheless, they appeared to correlate with the concentrations of Br, Mg, and Sr, which merits further investigation using larger sample sizes and higher variabilities of the ecological state of mussels.

**Abstract:**

A both wild and farmed mussels in natural conditions, anthropogenic inputs are usually reflected in the increase of the content of specific elements. To determine the possible effect of the elemental patterns of farmed and wild mussels (*Mytilus galloprovincialis*) collected in the Saldanha Bay area (South Africa) on the crystallographic texture of the shells, the content of 20 elements in shells and 24 in the soft tissue of mussels was determined by neutron activation analysis. The crystallographic texture of mussel shells was analyzed using time-of-flight neutron diffraction. The wild mussels from open ocean site live in stressful natural conditions and contain higher amounts of the majority of determined elements in comparison with mussels farmed in closed water areas with anthropogenic loadings. The changes between the maximums of the same pole figures of the three samples are in the range of variability identified for the genus *Mytilus*. The content of Cl, Sr, and I was the highest in mussels from the open ocean site, which is reflected by the lowest mass/length ratio. The determined crystallographic textures of mussels are relatively stable as shown in the analyzed pole figures despite the concentrations of Na, Mg, Cl, Br, Sr, and I in shells, which significantly differ for wild and farmed mussels. The stability of the crystallographic texture that we observed suggests that it can be used as a reference model, where if a very different texture is determined, increased attention to the ecological situation should be paid.

## 1. Introduction

*Mytilus galloprovincialis*, a cosmopolite mussel species, has a wide geographical distribution spanning from the Subtropics to the Arctic. In addition, among other species of mussel (including filum *Mytilus*), it tolerates changes of a wide range of environmental parameters (temperature, salinity, and pH). The adaptation potential *of Mytilus galloprovincialis* is reflected in the high ranges of micro- and macroelement concentrations relative to the anthropogenic or natural inputs.

The number of works focused on the content of elements in shells of mussels is limited. Usually, the relationships between potential elemental proxies by using specific ratios (element/Ca or element/Na) or effect of environmental factors such as temperature [1], salinity, pH [2,3], and seawater chemistry on elemental composition is investigated. Some studies have focused on the analysis of micro or macroelements along transects of shells to describe the temporal trends connected with change of the environmental parameters and growth rates [1]. Several works are dedicated to the analysis of the interlinking of elements in soft tissues and shells of mussels used as bioindicators of water pollution [4,5]. However, in such studies, the number of determined elements is usually low (3–12 elements). They are hindered by the limits of detection of the techniques and lack of reference data about microelement concentrations.

Since bivalves are sensitive organisms, it is assumed that environmental changes could affect the texture of their shells. The effect of salinity and pH treatments on the crystal orientation in mussel shells was studied by Grenier [6]. They showed that biomineralization processes are significantly affected by lower pH conditions than by lower salinity. It is important to note that in real estuarine conditions, mussels are not exposed to such low levels of salinity and pH to reveal the adaptation mechanism.

The present study aimed to determine the elemental patterns of farmed and wild mussels collected in the Saldanha Bay area (South Africa) and to assess their possible effect on the crystallographic texture of the shells. Neutron activation analysis (NAA) was applied to determine the elemental composition of the samples. The technique is used for an accurate determination of a large number of elements, including rare earth elements, heavy elements such as Sb, Cs, Th, U, and nonmetals such as As, Se in samples with different matrices [7].

Crystallographic texture studies are traditionally carried out using X-ray or electron backscattered diffraction (EBSD). However, due to the small penetration depth into the substance, it is impossible to obtain the necessary information for a complete description of the texture using these methods. Information about texture is traditionally obtained from pole figures. Complete pole figures can only be measured by thermal neutron diffraction. Moreover, it should be noticed that the X-ray and EBSD techniques have special requirements, in that a sample must be flat, whereas a sample for neutron measurements does not require special preparation. It is known in material science that adding elements can drastically change the crystallographic texture of alloys [8]. Therefore, it is of interest to study the question of whether the texture of mollusk shells changes with alteration in their elemental composition. Both techniques are non-destructive and characterized by high sensitivity.

For this study, the Saldanha Bay zone was chosen for its water areas with farmed and wild mussels. The Danger Bay is situated out of the Small bay and the Langebaan lagoon and is influenced by open ocean waters. These three zones have been characterized by a relatively uniform vertical structure of the water column based on temperature and salinity changes [9]. The Saldanha Bay Harbor is a developing industrial area, which exports such products as coal, lead, steel, titanium slag, zinc concentrate, and zircon [10]. These materials could be sources of water pollution with chemical elements, which could accumulate in marine bivalves growing in nearby zones. However, according to ecological state reports, the concentrations of trace elements in mussels from the studied area decreased in the last decade [9]. In mussels from the Saldanha area, the levels of elements were analyzed by several authors [11,12], including our works [13,14,15,16].

Metal concentrations in mussels *Mytilus galloprovincialis* had been measured since 1985 as part of the Mussel Watch Programme [17]. which. provides some indication of bioavailable metals in the coastal environment. The lack of data on metal concentrations in shells in addition to soft tissues and established limits of their dangerous or safe levels in natural conditions increase the importance of such studies.

## 2. Materials and Methods

### 2.1. Sampling

**Figure 1:**
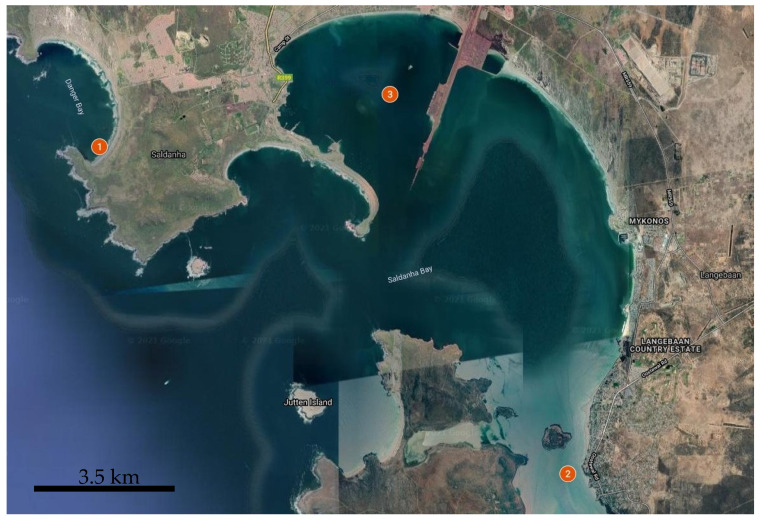
Collection sites in Saldanha Municipality area: St. 1—Danger Bay, St. 2—Langebaan Yacht club, St. 3—Small bay.

The samples of mussels were collected from three stations in the Saldanha Bay from April 16 to May 09, 2019, before the spawning period (Figure 1). The stations were chosen as the most representative in terms of availability of mussels, stability of environmental parameters, and differences in pollution level according to previous studies [13,14,15,16]:

St. 1. Danger Bay was chosen to represent the features of wild mussels, which grow in a pristine tidal zone and are exposed to waves and sunlight.

St. 2. Mussels from Langebaan Yacht Club were grown under the jetty and were always submerged in water. This site is close to the West Coast National Park, exposed to pollution sources at the mouth of the lagoon.

St. 3. Mussels in Small bay were grown at the West Coast Aqua Culture farm.

From each site, 11–17 individuals of one typical size (50–70 mm) of *Mytilus galloprovincialis* were chosen randomly and collected. The number of mussels was determined with the following considerations: minimum number of individuals necessary per sampling site, representativeness of the subsample, and minimization of the deviations within the group. In addition, the collection of such number of mussels does not harm the natural population.

The length (maximum anterior-posterior axis), height (maximum dorsal-ventral axis), and width (maximum lateral axis) of shells were measured according to Lauzon-Guay et al. [18]. For biometric characterization, the means of the following ratios were calculated and presented further: mass of soft tissue (mg/kg, dry weight)/length (mm); weight (mm)/height (mm); and conditional index (mg/cm^3^) calculated as mass of soft tissue (dry weight)/length × width × height, according to [19].

The samples were frozen and transported to the Joint Institute for Nuclear Research, Dubna (Russian Federation) for further analysis.

### 2.2. Characterization of the Environmental Parameters

The temperature and salinity were in the typical range for open ocean zones according to the Saldanha bay state report [9]. They ranged from 13 to 20 °C and from 32 to 35 PSU, respectively, independently of rain and tidal activities. Even rain and weather changes influencing one of the sites more than the others, the salinity and temperature fluctuations were not as great. This resulted in the adaptation of mussels to specific hydrophysical conditions.

Salinity remained constant within a narrow range for the studied period (34.6–34.9 PSU). High or low salinity outliers were not detected during the monitoring program in the April-May season [9].

In general, at all key sites of the bay during the monitoring study, the water profiles were found to be consistent. Bottom water with lower salinity usually enters the Bay during the upwelling season (summer), and high salinity surface waters are developed in late summer/autumn (the study period).

The introduction of oceanic waters into Saldanha Bay could be identified based on temperature and salinity measurements and it results in the reduction of the levels of nitrate and chlorophyll concentrations. Rarely the fast intrusions in the bay (St. 2 and 3) could change the relatively stable stratification in the upper layer. It tends to create stress conditions for mussels which are expressed by increased rates of accumulation of the majority of microelements in the soft tissue of the organisms. St. 1 is situated in a zone constantly open to the influence of ocean waters and storm activities, so the mussels from this station grow in more stress-related conditions than mussels from the other sites.

Usually, the dissolved oxygen in the bottom waters of the bay reaches low levels in the summer and autumn due to storms and vertical mixing of the water column. The alternate stratification and water column mixing associated with upwelling and relaxation phases over 3–10 day periods are set in the autumn period [9]. The extremely low dissolved oxygen values were recorded for a short period in May (1 to 2 mg/L) which were below the tolerable levels for invertebrates and fish [9]. According to Clark, such low oxygen events could be associated with an introduction of cold water from the adjacent coast where low oxygen water is known to occur during autumn.

Turbidity in the Saldanha Bay usually increases under strong wind conditions (due to the wind and wave action that suspends particulate matter in the water column) and the Danger bay is highly exposed to this impact [9]. Langebaan Lagoon, however, typically remains clear with low levels of turbidity even when the winds stay strong. This could be explained by the coarse structure of the sediment in the lagoon compared to the finer sediment in Saldanha Bay [9]. The water column turbidity data reflected the same general trends, the turbidity in winter generally ranged from 5 to 12 NTU, and in other seasons it typically falls within the interval between 5 and 8 NTU [20].

The influx of nutrient-rich upwelled water into Saldanha Bay is a critical factor for the growth of natural mussel assemblages or mariculture.

The fact that the thermocline is shallower than 5 m depth means that the shallower parts of Saldanha Bay, particularly the Langebaan Lagoon, are not exposed to nutrients (mainly nitrate) from the Benguela upwelling system [9]. As a result, these shallow water areas do not support large plankton blooms and are usually clear.

It is known that winds and upwellings tend to create stress conditions for the mussel population. The stratification of the water column from spring to summer can be caused by wind-driven upwelling, and during the winter the water column stays more or less isothermal [9]. Earlier continuous monitoring of the temperature identified a three-week break in the usual upwelling cycle in December 1999, with consequent gradual warming of the bottom water. This event was associated with a decrease in phytoplankton production due to reduced import of nutrients, which negatively affected local mussel mariculture [21]. Sampling for this study was performed in April-May, as relatively stable stratification conditions were assumed based on the monitoring study by [21].

### 2.3. Elemental Analysis

In the laboratory, mussels were rinsed with deionized water to remove mud, sediments, and other particles. For NAA each mussel was dissected with a plastic knife, separated into the shell and soft tissue batches, and weighed. The material of soft tissues and shells was firstly lyophilized and then dried to constant weight at 105 °C.

To decrease the inter-individual variability, which was previously studied [14,16], the soft tissues and shells of all mussels from each site were grouped into pooled samples and homogenized.

The samples were homogenized to powder using a planetary mono mill with agate milling balls (PULVERISETTE 6, Fritsch Laboratory Instruments GmbH, Germany) at 400 rpm. Three repeats for each station were created and analyzed independently. The 0.3 g of sample were packed into polythene bags for the determination of short-lived isotopes and aluminum cups for long-lived isotopes of elements.

Neutron activation analysis at the REGATA facility of the reactor IBR-2 (Dubna, Russian Federation) was applied for the determination of 23 macro- and microelements in soft tissues and 20 in shells for each pooled batch of mussels. This method fits well for the determination of specific groups of elements considered as markers, key elements, which allow for estimation of the significance of terrigenous and anthropogenic factors [16].

The instrumental neutron activation analysis was performed differently for 3 groups of elements according to the following determination types [7,16]. For short-lived isotopes (Mg, Al, S, Cl, Ca, Ti, V, Mn, I) the subsamples were irradiated at a neutron flux of 1.6 × 10^12^ n cm^−2^ s^−1^ for 3 min and immediately measured for 15 min. For long-lived isotopes, the subsamples were irradiated with epithermal neutrons in a channel with a cadmium shield at a neutron flux of 3.31 × 10^11^ n cm^−2^ s^−1^ for 3 days, after 3 and 20 days of decay samples were measured for 30 min (for such elements as Na, K, As, Br, and U) and 90 min (for Sc, Cr, Fe, Co, Ni, Zn, Se, Rb, Sr, Sb, Cs, and Th), respectively. The spectra of induced gamma activity were measured with HPGe detectors (Canberra) with a resolution of 1.9 keV at 1332 keV total absorption peak of ^60^Co. The detector is calibrated using standard spectrometric and certified reference materials [22].

Specialized software developed in FLNP JINR [21] was used to create a Group Standard Sample (GSS) from standard reference materials (SRMs) irradiated simultaneously with the samples to calculate the content of elements with maximum accuracy. The GSS is also used to test the quality of the analysis. This procedure, applied to SRMs, allowed to compare the obtained and certified values and provided quality control of the analysis [7].

For quality control assurance (Table 1), standard reference materials (SRMs) of different origin provided by the National Institute of Standards and Technology (NIST), Institute of Nuclear Chemistry and Technology (INCT/ICHTJ) and Joint Research Centre (JRC), Institute for Reference Materials and Measurements (IRMM) were used: JRC-IRMM-BCR-063R (Skim milk powder), NIST1633c (Coal fly ash), NIST1549 (Non-fat milk powder), NIST1632d (Trace elements in coal), NIST2710a (Montana Soil), NIST2711a (Montana soil), INCT-CTA-FFA1 (Fine fly ash), JRC-IRMM-BCR-667 (Estuarine sediment), and NIST1566b (Oyster tissue). For the majority of elements, the concentrations were in the range of 5% deviation between determined and certified values. Since the standard material for oyster tissue (1566b) contains only a limited number of certified values, the use of standards of different matrices allowed to expand the number of determinable elements in the mussel samples, [16]. Chemical matrix effects, known to be significant sources of error in other types of instrumental chemical analysis, are insignificant in NAA. In the case of small samples (size and weight), the use of reference materials with matrices different from the analyzed sample is provided by the insignificant matrix effect in NAA [23].

### 2.4. Crystallographic Texture Analysis of Minerals of Shells

The pole figures presented in this work were measured at the Frank Laboratory of Neutron Physics of the Joint Institute for Nuclear Research (Dubna, Russia). For that, the SKAT spectrometer (Spectrometer for Quantitative Texture Analysis) located on channel 7A-2 of the IBR-2 pulsed nuclear reactor was used [24]. Time-of-flight neutron diffraction was used to obtain diffraction reflections corresponding to the minerals that make up the shells.

SKAT consists of a detector ring (2 m diameter), on which 19 detector-collimator complexes are located at the same scattering angle 2*θ* = 90°. The detector modules are mounted on the circumference of the detector ring in such a way that the step on the pole figure is 5°. The long flight path of more than 100 m and the collimator system provide a relatively high resolution of Δd∕d = 5 × 10^−3^ at d = 2.5 Å and 2*θ* as reported by Keppler et al. [25]. This resolution allows having non-overlapped diffraction peaks for some of the calcite and aragonite reflexes of which the shells are composed. Pole figures are extracted from measured 19 × 72 = 1368 diffraction patterns that correspond to the 5° × 5°. It should be noted that thanks to the time-of-flight technique, pole figures from all minerals (phases) presented in the sample are measured simultaneously, that is, they are extracted from the same patterns. The cross-section of the neutron beam of 50 × 90 mm makes it possible to measure large samples up to 100 cm^3^. It should be emphasized that each partial pattern was measured under the same conditions, meaning that the pole figures obtained in this way do not need correction. Furthermore, due to the large penetration depth of neutrons, bulk samples of centimeter size can readily be investigated in transmission geometry.

The most intense isolated diffraction reflections in each of the recorded patterns were analyzed using the Pole Figure Extractor program [26] to determine the nature of the distribution of the corresponding crystallographic planes in the valves. The intensity of one reflection corresponding to the crystallographic plane with certain Miller indices gives one point on the pole figure indicated by these indices. The conventional approach to extract pole figures from the measured diffraction patterns was used. The way to assign integrated intensity and background value to the corresponding pole figure point could be summarized by formulas:

(1) $$II(\vec{y})=(t_{\max}-t_{\min})\sum_{i=t_{\min}}^{i=t_{\max}}I(t_{i}),\;Bkg(\vec{y})=\frac{1}{\left(t_{\max}-t_{\min}\right)}\sum_{i=t_{\min}}^{i=t_{\max}}I(t_{i}).$$

In this case, a part of the measured pattern containing the diffraction reflex is divided into three regions. The side regions are used for background estimation whereas the central region is for intensity estimation.

The intensity on the pole figure is:

(2) $$P_{h_{i}}(\vec{y})=II(\vec{y})-\frac{Bkg_{left}(\vec{y})+Bkg_{right}(\vec{y})}{2}.$$

The approach for PF extraction based on a summation of the intensities was selected because the peak/noise ratio for both calcite and aragonite phases was not very high. It was revealed that the accuracy of pole figures extraction depends on peak/noise ratio [27]. All extracted pole figures were normalized and smoothed with the same parameter [28].

The more ordered the mineral crystals the higher the intensity on the pole figure (pole density), expressed in units of isotropic distribution (multiple random distribution, MRD). An increase of pole figure intensity is interpreted as texture strengthening. A pole density value equal to unity means that the corresponding crystallographic planes are uniformly distributed in all directions in the sample.

**Figure 2:**
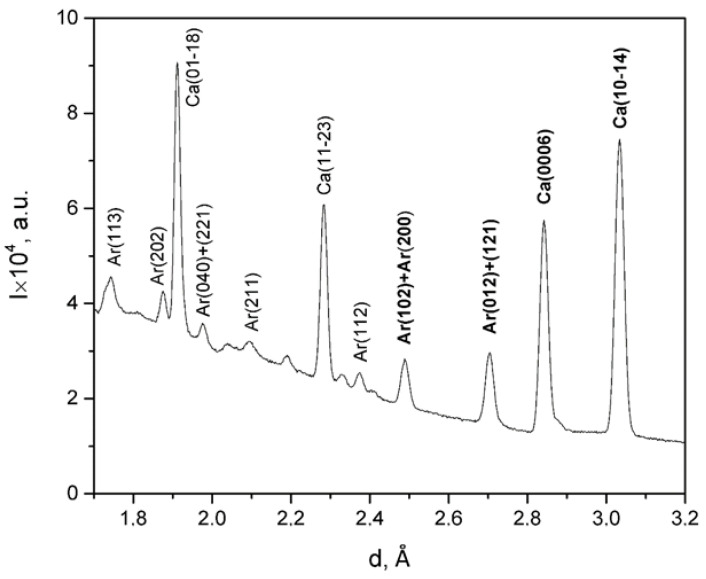
The sum diffraction pattern for mussel shells from station 2 (Langebaan).

For the study, valve sizes of at least 30 mm in length and a mass of at least 10 g were selected. The 3–4 valves were connected using a two-component adhesive. This was carried out to collect a volume of material sufficient for measurements. Then the sample prepared in this way was attached to a glass pin and fixed in the goniometer of the setup (Figure 2). The measurement time for each sample was 22 h.

Either only the left or only the right valves were used. They were studied as a whole, and not as valves surface fragments as is the case of backscattered electron diffraction or X-ray texture analysis.

The shells consist of two mineral phases which are calcite and aragonite. The most intense diffraction reflections corresponding to crystallographic planes with Miller indices (0006) and (10–14) for calcite and (012)/(121) and (102)/(200) for aragonite were analyzed. The neutron diffraction pattern for one of the samples is presented in Figure 2, and it is the sum of 1368 partial patterns measured for different directions.

### 2.5. Statistics

The obtained data were checked and analyzed by STATISTICA 12 software. For descriptive statistics, the means and standard deviations, excluding the outliers were used. For an indication of the precision of concentrations, we were used the coefficients of variations (CV = SD/mean) in %. For assessment of the significance of differences between the levels of mean concentrations among stations, the Kruskal-Wallis test [29] was performed for the following reasons: the small number of observations (sample size), normality of the data is nonessential (a non-parametric test), each observation is independent of others.

## 3. Results and Discussion

### 3.1. Elemental Content of Shells and Soft Tissues and Allometry

Concentrations of 20 elements determined in shells and 24 elements—in soft tissues of mussels from 3 stations are presented in Table 2. According to obtained data, the shells of *Mytilus galloprovincialis*, as well as the soft tissues, were good accumulators of elements such as Na, Mg, Al, Cl, Sc, V, Cr, Mn, Fe, Co, Ni, Zn, Br, Sb, I, Cs, Th and U. However, the low levels of concentrations for majority of analyzed elements decrease the abilities to use shells in biomonitoring studies. It is worth to note that levels of such elements as Sc, Sb, I, Cs, and Th were found in shells close to those in soft tissues, which means that shells could be used as biomonitors for such elements.

For comparative assessment, the mean levels of elements among stations and literature data are presented for shells in Table 3 and soft tissues in Table 4.

The significant differences between stations were found for the following elements:

In shells:

The concentrations of Cl, Sr, I in mussels from st. 1 were significantly higher (Kruskal-Wallis test, *p* < 0.003, *p* < 0.02, and *p* < 0.05, respectively) than in mussels from St. 2 and 3. The highest concentrations of Na and Mg were found at St. 2 (Langebaan) and they significantly differ (Kruskal-Wallis test, *p* < 0.01 and *p* < 0.02, respectively) from St. 1 and St. 3. The highest concentrations of Br were found at St. 3 and it significantly differ (Kruskal-Wallis test, *p* < 0.03) from St. 2 and St. 3.

In soft tissues:

The highest concentrations of Na, Mg, Cl, Cr, Fe, Co, Ni, Zn, As, Br, Sr, Sb, I, and U were determined at St. 1 and they significantly differ (Kruskal-Wallis test, *p* < 0.002 to *p* < 0.03) from St. 2 and 3. The highest concentrations of As, Se, Rb, Cs, and Th at St. 2 significantly differ (Kruskal-Wallis test, *p* < 0.004 to *p* < 0.03) from St. 1 and 3. The majority of elements (except Zn, As, Se, and Rb) were significantly lower (Kruskal-Wallis test, *p* < 0.01 to *p* < 0.004) at St. 3 than at St. 1 and 2.

The levels of elements in shells from the Saldanha zone are in agreement with similar data for mussels from the Adriatic area (Table 3). However, the concentrations determined by Fatoki et al. [4] in mussels collected in Cape Town were higher than in mussels from Saldanha bay according to our study. They analyzed large mussels of *Mytilus galloprovincialis* (50–70 mm), using two different techniques: EDXRF and ICP-MS methods. The high concentrations are probably connected with the contribution of terrigenous and anthropogenic components expressed by the high levels of such elements as Al, V, Cr, Mn, Fe, Co, Ni, Zn. The concentrations of these elements in [4] were greater than in mussels from Saldanha bay by one order of magnitude. On the other hand, our data revealed higher levels of Ca and Sr.

The data from the Mediterranean, Cape Town, and other sites in the Saldanha Bay area were used for the comparison of levels of elements in soft tissues of mussels [4,16,30,31,32,33] (Table 4) by using a review study of Yap et al. [34]. Our data were in agreement with the ranges of selected elements in Mytilus galloprovincialis from Spain [31], and the Mediterrainean water area [30,32]. The exception corresponded to mussels from the impacted area in the study of Signa et al. [32]. The individuals from Strandloper, which is situated in the greywater outlet in the Saldanha zone, contained elements at similar or slightly lower levels than those of St. 1 (Danger Bay). It could be explained by an established tolerance of mussels from St. 1 (Danger bay) to stress events and the regulation of levels of elements in soft tissues during the process of adaptation at this station. However, for the majority of elements in mussels, the levels from the polluted zone of Cape Town, as presented by Fatoki et al. [4], and the maximal levels in a study by Richir et al. [33] were higher than those obtained in the present study.

The levels of elements in soft tissues could be compared with maximum permissible levels established for seafood products (MPL) in wet weight (ww) [16,35,36]. For this purpose, the obtained concentrations of elements were transformed from dry to wet weight by a conversion factor (wet weight/dry weight = 0.32), which was calculated for the Saldanha zone previously [16].

The average levels of MPLs for Cr, Ni, Zn, As, and Se were set as 1, 80, 200, 3, and 1.2 µg/g, respectively (according to [35,36]). The mean concentrations of Cr (1.1 µg/g, ww) in the soft tissue of the studied mussels from St. 1 (Danger Bay) exceeded the average MPL probably due to the presence of suspended material with high content of Cr. This is also in agreement with a higher concentration of Cr in shells from St. 1 in respect to other stations. The average MPL for Se (1.2 ppm, ww) exceeded the set point value in soft tissues of mussels from all studied stations (1.6–2.3 ppm, ww). The values for Mn, Ni, Zn, and As, which are known to indicate pollution features, were below the average MPLs in mussels from all stations. Such levels of elements reveal the non-polluted state of mussels collected in the autumn of 2019.

Shells undergo erosion depending on the weather, the position of individuals within the mussel bed and on a substrate, etc. Thus, all of these parameters can hypothetically affect the structure of the shell.

The width/height (W/H) ratio could be used as an indicator of both the relative age and growth rate for the mussels [19]. A high width/height ratio indicates individuals with decreasing growth rate and increased relative physiological age. Moreover, this ratio is an important factor usually associated with high element concentrations [19]. For young fast-growing mussels, the W/H ratio at all sites was 0.6–0.8 while for older mussels of any length it was in the range of 1.0–1.1. The W/H ratio was the highest at St. 1 (0.94, Table 5), and the lowest at St. 2 (0.69).

In addition to indicating the differences in features of growth, the width/height ratio is an important factor associated with element concentrations. It has been reposted that increased element concentrations are associated with decreased growth rate [19]. For mussels from St. 1 (Danger bay) having a higher width/height ratio and a lower mass/length ratio, the highest levels of the majority of micro and macroelements were obtained. However, the condition index was at the level of mussels from St. 2 with lower levels of elements. This could be explained by the action of the stress factors at the Langebaan yacht club (St. 2) such as coastal pollution and ship traffic.

The maximum length of mussels varies from site to site and is generally associated with nutritional factors (e.g., mussels on the upper shore are much smaller than subtidal mussels) but also with stress (e.g., wave action, pollution). The mass/length ratio could reflect the processes of degradation of individuals expressed in decreasing the mass of soft tissues and increasing the length and width of shells [19]. The mass of soft tissue could fluctuate and may even decline with senescence [19] that agreed with W/H ratios and growth features.

According to the low values of the mass/length ratio, mussels from Danger Bay have the lowest weight of soft tissue per shell size. They are exposed to the open ocean, so rapid and continuous changes in wave activity can cause stress to these organisms. This process affects allometry parameters and, according to our data, is reflected in high concentrations of such elements such as Na, Mg, Cl, V, Cr, Fe, Co, Ni, Zn, As, Br, Sr, Sb, I, and U in soft tissues and Al, Cl, Cr, Fe, Ni, Zn, Sr, and I in shells.

However, the conditional index calculated from allometry parameters of shells and mass of soft tissues showed that the mussels from St. 1 grow in better or the same conditions as at St. 2 (Langebaan). That could be explained by the adaptation of individuals to stress conditions at St. 1 On the other hand, the mussels from St. 2 can be are exposed to stress associated with suspended particles of terrigenous origin, which is expressed in high concentrations of elements such as Al, Sc, Rb, Cs, Th (markers of a terrigenous component) in soft tissues. The mussels from St. 3 (Small bay) revealed the highest conditional index according to their morphometry parameters. It is in agreement with the lowest concentrations for the majority of elements in soft tissues determined at this site.

### 3.2. Environmental Features Based on Element/Na and Element/Ca Ratios

The highest levels of the majority of elements (except Al, Cl, Zn, Sr, and I) in soft tissues of mussels were determined in samples from St. 1 (Danger Bay). This revealed the specific state of mussels from this zone, exposed to the storms, tidal activities, and impact of open ocean waters. Consequently, they accumulated elements such as Na, Mg, Br are in concentrations significantly higher (Kruskal-Wallis test, *p* < 0.03) than at the other stations, which can be explained by the higher concentrations of elements in the water at St. 1. In addition, this could be connected with the high growth rates of farmed mussels (at St. 3) in comparison with wild offshore mussels [10]. Another possible explanation is the early spawn period in mussels at St. 1 because the trace metal accumulation rates have been linked to the gametogenic cycle [12]. Our previous studies also revealed higher levels of the majority of elements in soft tissues of mussels at this station [13,15]. The specific state of the mussels is expressed also in the lower weight of the soft tissue and greater length of the shells (described further). It is interesting to note that the levels of elements in shells from this station were lower than from other places. Thus, the soft tissues accumulate high levels of micro and macroelements and subsequently release them into surrounding waters, and do not incorporate them into their shells, except for Al, Cl, Zn, Sr, and I, which probably correspond to with their concentration in water.

Sodium was used as a salinity proxy for the determination of elements that may originate from marine waters. Salinity was reflected in the soft tissue of mussels in the following manner: the ratios Element/Na for K, Ca, Mn, Se, Rb were in the increasing order: at St. 1 (Danger Bay), St. 2 (Langebaan), St. 3 (Small Bay). In shells, the ratio of Br/Na is distributed in a similar order (St. 1, St. 2, St. 3). However, the ratios Element/Na for the rest of the determined elements revealed that the shells of mussels from St. 2 (Langebaan) contain lower levels than in mussels from St. 1 (Danger bay), yet higher than mussels from St. 3 (Small bay).

Calcium as a matrix element in shells is present in the form of calcite or aragonite minerals. The ratios Element/Ca reveal elemental impurities in the shell structure. The high content of elements such as Al, Cl, Cr, Fe, etc. in shells of mussels from St. 1 resulted in high values of the Element/Ca ratios. However, these elements are not considered anthropogenic, since the station is situated in a pristine area.

Mg/Ca is considered as a temperature proxy in shells of mussels (*Mytilus edulis* and *Mytilus trossulus*) [1]. The values of the Mg/Ca ratio in mussels from St. 2 (Langebaan) were higher than from St. 1 and 3, which agrees with the higher temperature of waters, which is characteristic of the Langebaan lagoon.

In mussels from St. 3 (Small bay) the Mn/Ca ratios reached lower levels than in mussels from St. 1 and St. 2, which can be associated with relatively low levels of nutrients and microalgae. It is known that high Mn/Ca levels could be associated with phytoplankton blooms [37]. The Mn/Ca ratio in many cases indicates chlorophyll in surrounding waters and is associated with the growth of phytoplankton assemblages, on which mussels feed [38].

### 3.3. Crystallographic Texture Results

Crystallographic texture is the preferred orientation of the crystallites composing a polycrystal. The information about the crystallographic texture is presented using pole figures [39]. A pole figure of polycrystalline material is the projection of the normals to a given atomic plane constructed for all crystals of the sample. The pole figure function $P_{\vec{h}}(\vec{y})$ gives the volume fraction of the sample for which the lattice plane normal $\vec{h}$ falls in various sample directions *y*. If the sample is isotropic (no texture), then the outlets of the plane normals uniformly cover the sphere. In a textured sample, the distribution of normals to the plane is irregular and is represented by lines of constant intensity (isolines). If such a distribution is projected onto the equatorial plane of the sphere, then this stereographic projection will be a pole figure.

**Figure 3:**
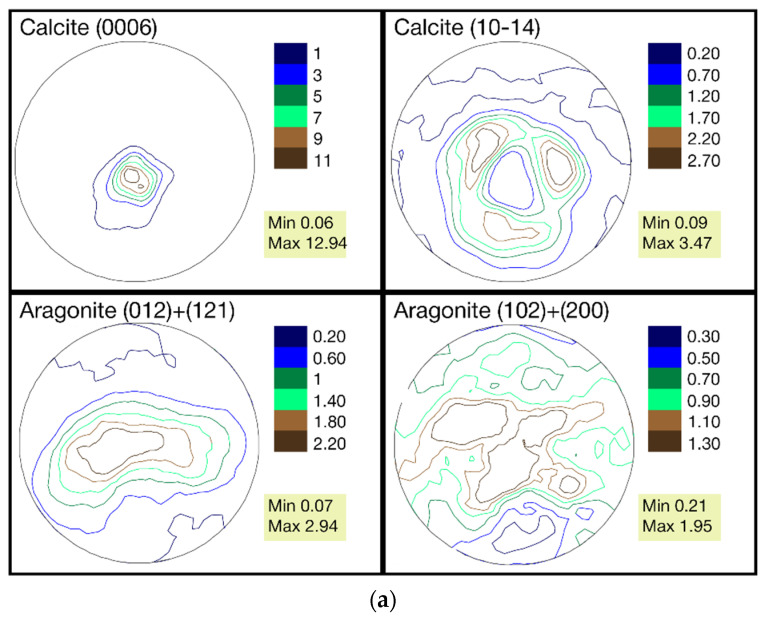

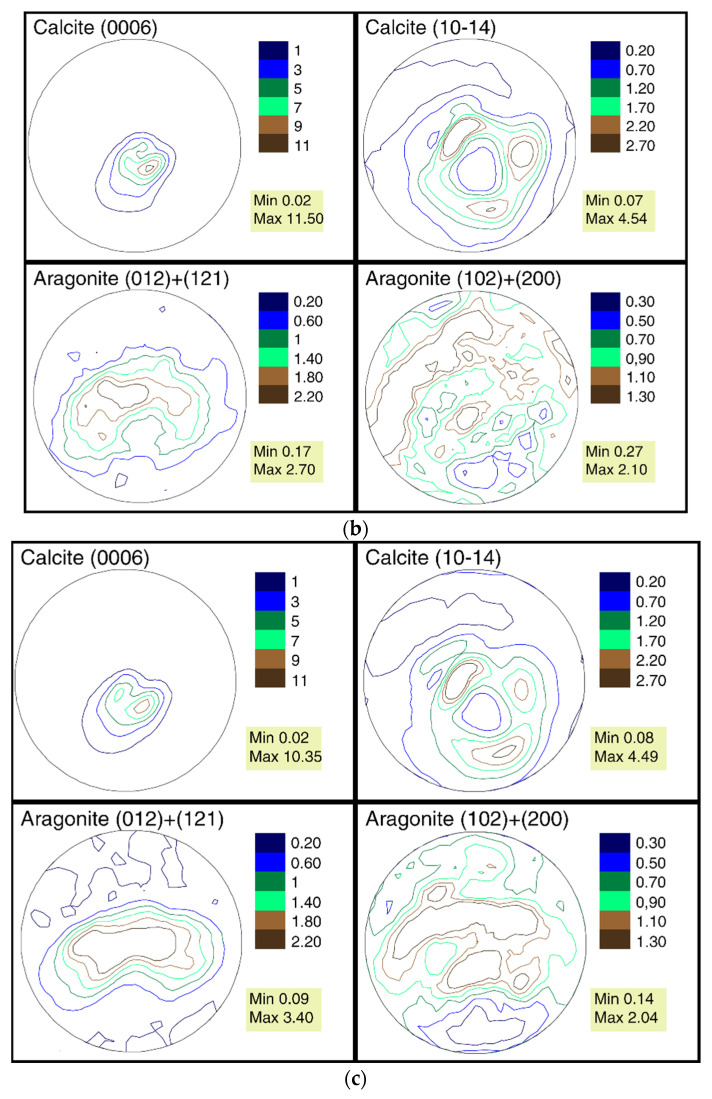
(**a**) The pole figures of calcite and aragonite phases for mussel shells from station 1 (Danger Bay). (**b**) The pole figures of calcite and aragonite phases for mussel shells from station 2 (Langebaan). (**c**) The pole figures of calcite and aragonite phases for mussel shells from station 3 (Small Bay).

The pole figures of the calcite and aragonite phases for samples collected from the three stations are presented in Figure 3a–c.

The calcite phase for all three samples has a very sharp texture, in contrast to the relatively weak texture of aragonite. Indeed, the maxima of pole density for the (0006) calcite pole figure is in the range of 10.35–12.94 MRD for these samples, whereas the ones for the (012) + (121) aragonite pole figure is in the range of 2.7–3.4 MRD. We analyzed the aragonite pole figures for two reflexes jointly because they are overlapped in the diffraction pattern. A similar crystallographic texture for calcite and aragonite phases for *Mytilus galloprovincialis* shells from Black and Azov seas was observed in previous investigations [24].

When comparing the pole figures of all three samples, it can be seen that the distribution of the texture components for both phases is practically the same. Nevertheless, quantitative changes in the pole density are noticeable. From a comparison of the maximums in the calcite sharpest pole figure (0006), it can be seen that the first sample with a maximum at 12.94 MRD is closer to the second one (11.5 MRD) than to the third (10.35 MRD). The fact that such a trend is not observed for the second calcite pole figure (10–14) and both aragonite pole figures can be explained by measurement errors, that, at the pole density maximum, are approximately 0.2 MRD. Furthermore, the changes between the maximums of the same pole figures of the three samples are in the range of variability identified for the genus *Mytilus* [24].

It should be mentioned that in quantitative texture analysis, the *RP* value is commonly used to characterize the similarities of the pole figures [40],

(3) $$RP_{i}=\sum_{j=1}^{J}100\%\Theta [\varepsilon ,P_{{\vec{h}}_{i}}({\vec{y}}_{j})]r_{ij}/\sum_{j=1}^{J}\Theta [\varepsilon ,P_{{\vec{h}}_{i}}({\vec{y}}_{j})]$$

where,

$$r_{ij}=\left|P_{{\vec{h}}_{i}}^{1}({\vec{y}}_{j})-P_{{\vec{h}}_{i}}^{2}({\vec{y}}_{j})\right|/P_{{\vec{h}}_{i}}^{1}({\vec{y}}_{j}),\;\Theta (\varepsilon ,\;x)=\left\{ \begin{array}{l} 0,x<\varepsilon \\ 1,x\geq \varepsilon \end{array}\right..$$

The results of the poll figures comparison between the samples using the *RP* values are presented in Table 6.

As can be seen from Table 6, the RP values between samples 1 and 3 are higher than between samples 1 and 2 for both calcite pole figures and one of the aragonite pole figures. However, this trend is not the same for the second aragonite pole figure. This implies no significant difference between the pole figures of the samples collected at the three stations.

**Figure 4:**
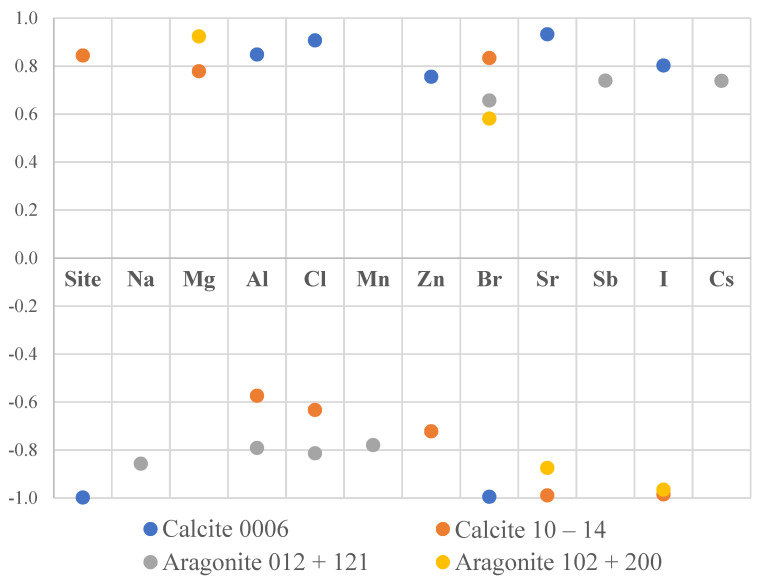
Significant correlations (*p* < 0.05) between the maximums of the pole figures for different phases and levels of elements.

An attempt was made to quantitatively study the texture sharpness with the concentration of various elements in the mussel shells. The correlation coefficients between the maximum values of the pole figures for different phases and the concentrations of the elements in shells are the following (Figure 4):

- Calcite (0006) correlated with Al, Cl, Zn, Sr, I (*r* > 0.75, *p* < 0.02) and inversely correlated with Br (*r* < −0.8, *p* < 0.001).
- Calcite (10–14) correlated with Mg, Br (*r* > 0.75, *p* < 0.03) and inversely correlated with Zn, Sr, I (*r* < −0.7, *p* < 0.3).
- Aragonite (012) + (121) correlated with Sb, Cs (*r* > 0.7, *p* < 0.02) and inversely correlated with Na, Al, Cl, Mn (*r* < −07, *p* < 0.01).
- Aragonite (102) + (200) correlated with Mg (*r* > 0.9, *p* < 0.001) and inversely correlated with Sr, I (*r* < −0.8, *p* < 0.002).

The small data set does not allow us to draw conclusions based on these results and further investigations are necessary for clarification of these trends.

## 4. Conclusions

The mussels are organisms suitable for long-term biomonitoring, they accumulate elements in typical ranges proportionate to the presence of elements in the surrounding waters. Thus, extremely short-term changes in the hydrophysical parameters such as temperature, salinity, or dissolved oxygen cannot be investigated following a period of stabilization. On other hand, coastal runoff and repeated long-time introductions of open ocean waters into coastal areas could affect the elemental and crystallographic structure of mussels. In this study, the determined ranges of concentrations of elements showed that mussels from the three studied areas were growing in non-polluted or natural stress conditions, which was ascertained by comparisons with data from polluted sites and maximum permissible levels for elements in seafood products (soft tissues).

The mussels from station 1 (Danger Bay) differ from mussels from another two sites in their concentrations of the majority of elements (in soft tissues and shells) and also in morphometry parameters. As wild organisms, mussels were adapted to the stress influences of the open ocean waters by increased width/height and decreased mass/length ratios.

Even though significant differences between concentrations of elements among the stations were found, the crystallographic textures of mussel shells from the studied bays showed insignificant dissimilarities. The observed differences in the maximum values in the pole figures fell within in the range of variability identified for the genus *Mytilus*. Nevertheless, they appeared to correlate with the concentrations of Br, Mg, and Sr, which merits further investigation using larger sample sizes.

For future investigations, we propose to determine the content of elements such as Na, Mg, Cl, Br, Sr, and I in mussel shells and Na, Mg, Al, S, Cl, Sc, Cr, Fe, Co, Ni, Zn, As, Se, Br, Rb, Sr, Ag, Sb, I, Cs, Th, and U in soft tissues. The results could be used to establish possible hydrochemical parameters for controlled environments in which mussels are grown.

## Figures and Tables

**Table 1:** Quality control for neutron activation analysis.

Elements	Standard Reference Materials (SRMs)	Concentrations, ppm		Uncertainties, %		Deviation, %
		Determined	Certified	Determined	Certified	
Na	2711a	11,900	12,000	8.8	0.01	0.5
Mg	063R	1320	1263	7.6	2	4.6
Al	1633c	129,900	132,800	6.9	4.6	2.2
Cl	1632d	1150	1142	7.8	1	0.3
K	FFA1	23,420	22,000	11.3	30	6.4
Ca	2710a	9450	9640	18.9	4.7	1.9
Sc	667	13.6	13.7	5.3	5.1	0.4
V	1632d	23.8	23.7	5.7	0.4	0.1
Cr	2711a	53.2	52.3	11.5	6	1.7
Mn	1549	0.27	0.26	15	23.1	4.2
Fe	2711a	27,900	28,200	5.5	0.04	1.2
Co	2711a	10	9.9	5.6	2	1.1
Ni	667	126	128	10.3	7	1.4
Zn	667	177	175	4.5	7.4	0.9
As	2711a	102	107	6	5	4.8
Se	FFA1	4.4	4.6	39.1	30	3.3
Br	667	82.8	99.7	4.4	2.5	16.9
Rb	2711a	126	120	16.6	3	4.7
Sr	FFA1	252	250	11.3	5.2	0.8
Sb	FFA1	18.4	17.6	7.6	14.2	4.6
I	1549	3.1	3.4	15.3	0.6	7.4
Cs	667	7.9	7.8	4.3	9	0.8
Th	667	10.2	10	4.7	5	2.1
U	667	2.31	2.26	3.3	6.6	2.1

**Table 2:** The ranges of concentrations of elements in shells and soft tissues of *Mytilus galloprovincialis* in the Saldanha zone.

Elements	Shells						Soft Tissues					
	St. 1		St. 2		St. 3		St. 1		St. 2		St. 3	
	Min-Max	CV	Min-Max	CV	Min-Max	CV	Min-Max	CV	Min-Max	CV	Min-Max	CV
Na	0.4–0.46	7	0.46–0.50	4	0.38–0.40	3	5.1–5.4	3	3.8–3.9	1	1.96–2.01	1
Mg	770–840	5	1040–1150	5	860–960	6	7000–7600	4	5500–6500	8	2860–3270	7
Al	39–52	15	33–46	17	15–23	26	159–203	13	444–480	4	63–71	6
Cl	790–850	3.7	720–820	7.3	530–560	2.8	81,000–86,000	3	61,000–62,000	1	29,700–33,100	6
K	<DL		<DL		<DL		7400–8500	7	6700–10,400	22	8700–10,400	10
Ca, %	3.-37	2.8	33–38	7.1	37–39	2.6	1.02–1.16	7	1.15–1.22	3	0.69–0.79	7
Sc	0.04–0.07	39	0.03–0.04	11	0.04–0.07	28	0.037–0.061	25	0.12–0.13	7	0.018–0.033	32
V	0.2–0.3	18	0.2–0.3	20	0.3–0.3	7	1.25–1.41	6	0.62–1.46	49	0.66–0.9	16
Cr	1.2–1.9	23	0.9–1.6	28	1.5–1.7	7	3.2–3.8	9	2.4–2.6	5	1.1–2.6	45
Mn	1.57–2.2	17	1.74–2.16	11	1.37–1.5	5	3.76–5.7	25	5–6.6	16	3.42–5.4	26
Fe	112–207	31	78–119	21	84–146	27	421–470	6	390–420	4	99–136	17
Co	0.09–0.14	28	0.06–0.12	33	0.04–0.11	48	0.46–0.52	7	0.225–0.26	9	0.128–0.136	3
Ni	0.4–0.6	24	0.1–0.6	78	0.2–0.6	50	3.1–4.7	24	0.83–1.51	30	0.75–0.82	4
Zn	4–4.3	4	3–3.9	13	2.62–3.77	18	230–232	0.4	105–108	1.6	121–124	1.2
As	<DL		<DL		<DL		8.3–8.6	2	5.3–5.8	5	5.7–6.2	4
Se	<DL		<DL		<DL		4.9–5.2	3	7–7.2	1	5–5.3	3
Br	64–68	4	88–89	1	111–113	1	463–467	0.4	289–291	0.3	163–165	0.6
Rb							3.5–3.8	5	4.9–5.2	3	3.9–4.2	4
Sr	1500–1550	2	1090–1100	1	1030–1050	1	133–136	1	98–99	1	52–53	1
Sb	0.02–0.04	32	0.01–0.03	39	0.03–0.06	34	0.042–0.057	15	0.021–0.033	24	0.0145–0.0206	19
I	12–13	3	5–5	2	6–7	12	56–57	1	12.3–13.7	5	4.8–5.7	9
Cs	0.05–0.08	29	0.04–0.05	13	0.05–0.12	39	0.031–0.037	9	0.081–0.091	6	0.024–0.038	24
Th	0.05–0.09	26	0.05–0.06	12	0.05–0.1	33	0.05–0.05	0.1	0.106–0.109	1.6	0.024–0.094	28
U	0.11–0.17	25	0.04–0.06	29	0.04–0.17	58	0.68–0.97	19	0.19–0.36	31	0.27–0.31	7

CV, %—coefficient of variation in percentages; <DL—below detection limit.

**Table 3:** Elements’ concentrations in shells of mussels (ppm, dry weight).

Elements	Saldanha Zone			[4]	[5]
	Danger Bay	Langebaan	Small Bay	Cape Town	Adriatic
	Mean ± SD	Mean ± SD	Mean ± SD	Mean ± SE	Range
Na	4300 ± 300	4767 ± 210	3933 ± 120	-	2500–4000
Mg	797 ± 38	1100 ± 56	913 ± 50	-	800–1400
Al	45 ± 6.6	39 ± 6.6	18 ± 0.1	3730 ± 960	2.5–27
Cl	823 ± 30	757 ± 60	543 ± 20	-	-
Ca, %	36 ± 1	35.3 ± 2.5	38 ± 1	12.6 ± 1.2	35–37
Sc	0.05 ± 0.002	0.03 ± 0.004	0.06 ± 0.002	-	-
V	0.3 ± 0.04	0.2 ± 0.05	0.3 ± 0.02	3.9 ± 0.2	-
Cr	1.6 ± 0.4	1.3 ± 0.4	1.6 ± 0.1	8.4 ± 0.1	-
Mn	1.9 ± 0.3	2 ± 0.2	1.4 ± 0.1	27.7 ± 1.3	2–155
Fe	155 ± 25	98 ± 21	116 ± 31	1360 ± 140	15–550
Co	0.1 ± 0.03	0.09 ± 0.03	0.09 ± 0.04	0.72 ± 0.01	-
Ni	0.5 ± 0.12	0.3 ± 0.23	0.4 ± 0.21	3.56 ± 0.04	7–33
Zn	4 ± 0.2	3 ± 0.5	3 ± 0.6	142 ± 13	2.5–15
Br	67 ± 2	89 ± 1	112 ± 1	-	-
Sr	1523 ± 25	1093 ± 6	1043 ± 12	365 ± 7	500–800
Sb	0.03 ± 0.009	0.02 ± 0.008	0.04 ± 0.015	-	-
I	13 ± 0.4	5 ± 0.1	6 ± 0.8	-	-
Cs	0.06 ± 0.02	0.04 ± 0.01	0.1 ± 0.04	-	-
Th	0.07 ± 0.02	0.06 ± 0.01	0.08 ± 0.03	-	-
U	0.13 ± 0.03	0.05 ± 0.01	0.12 ± 0.07	-	-

SD—standard deviation; SE—standard error.

**Table 4:** Concentrations of elements in the soft tissue of *Mytilus galloprovincialis* (dry weight, ppm).

Elements	Out Study, Saldanha Zone	[30] Min-Max	[31] Min-Max	[32] Min-Max	[16] Mean ± SD	[33] Min-Max	[4] Mean ± SE
	Min-Max						
Na	19,600–54,000				29,550 ± 5350		
Mg	2860–7600				6960 ± 1040		
Al	63–480				134 ± 53	39–877	
Cl	29,700–86,000				6660 ± 1920		4330 ± 180
K	6700–10,400						3.69 ± 0.05
Ca, %	0.69–1.22				1.02 ± 0.70		
Sc	0.02–0.13				0.035 ± 0.014	2.3–12.5	3.3 ± 0.3
V	0.6–1.5			11–59	0.63 ± 0.24	0.19–2.17	5.9 ± 0.2
Cr	1.1–3.8		0.9–4.7	12–25	1.7 ± 0.4	3.4–20.6	23.2 ± 1.5
Mn	3.4–6.6			125–144	3.92 ± 0.99	66–656	1150 ± 150
Fe	99–470	98–685	52–215		171 ± 51	0.37–1.37	0.70 ± 0.02
Co	0.1–0.5			3–158	0.21 ± 0.03	0.71–3.39	1.78 ± 0.02
Ni	0.8–4.7		0.2–1.5	20–39	1.1 ± 0.24	35–224	204 ± 15
Zn	105–232	117–423	85–284	23–265	309 ± 105	17–46	9.2 ± 0.1
As	5.3–8.6			1.3–15.6	7.7 ± 1.6	1.5–4.3	
Se	4.9–7.2				2.7 ± 0.3		
Br	163–467	91–460			350 ± 58		
Rb	3.5–5.2				3.1 ± 0.52		209 ± 5
Sr	52–136	18–67			77 ± 50	0.007–0.028	
Sb	0.01–0.06				0.02 ± 0.005		
I	4.8–57				46.7 ± 8.2		
Cs	0.02–0.09				0.024 ± 0.009		
Th	0.02–0.11				0.034 ± 0.012		
U	0.2–1.0				0.4 ± 0.07		

CV (%)—coefficients of variations (min-max among studied sites); SD—standard deviation; SE—standard error.

**Table 5:** Morphometry ratios in mussel samples from 3 stations of Saldanha Bay.

Parameters	St. 1 Danger Bay	St. 2 Langebaan	St. 3 Small Bay
**Mass/Length**	0.03	0.05	0.09
**Width/Height**	0.96	0.69	0.84
**CI**	17.7	16.6	22.1

CI—condition index (mg/cm^3^) calculated as [Mass (g) × 10^6^]/[Length (mm) × Width (mm) × Height (mm)] [19]. The standard deviation for calculated ratios (*n* = 5) were less than 10% for each station.

**Table 6:** *RP* (%) values for calcite and aragonite pole figures for the samples of *M. galloprovincialis* shells.

Pole Figures (PF)	Calcite (0006)		Calcite (10–14)		Aragonite (012) + (121)		Aragonite (102) + (200)	
**Station**	2	3	2	3	2	3	2	3
1	32.7	38.3	9.1	14.7	7.2	14.4	5.0	2.9

## Data Availability

Not applicable.
